# TLR4, but Neither Dectin-1 nor Dectin-2, Participates in the Mollusk Hemocyanin-Induced Proinflammatory Effects in Antigen-Presenting Cells From Mammals

**DOI:** 10.3389/fimmu.2019.01136

**Published:** 2019-05-31

**Authors:** José M. Jiménez, Michelle L. Salazar, Sergio Arancibia, Javiera Villar, Fabián Salazar, Gordon D. Brown, Ed C. Lavelle, Luisa Martínez-Pomares, Jafet Ortiz-Quintero, Sergio Lavandero, Augusto Manubens, María Inés Becker

**Affiliations:** ^1^Fundación Ciencia y Tecnología Para el Desarrollo (FUCITED), Santiago, Chile; ^2^Aberdeen Fungal Group, Medical Research Council Centre for Medical Mycology, University of Aberdeen, Aberdeen, United Kingdom; ^3^School of Biochemistry and Immunology, Trinity Biomedical Sciences Institute, Trinity College Dublin, Dublin, Ireland; ^4^School of Life Sciences, University of Nottingham, Nottingham, United Kingdom; ^5^Facultad de Ciencias Químicas y Farmacéuticas, Facultad de Medicina, Advanced Center for Chronic Diseases, Universidad de Chile, Santiago, Chile; ^6^Biosonda Corporation, Santiago, Chile

**Keywords:** mollusk hemocyanins, antigen-presenting cells, inflammation, Dectin-1, Dectin-2, Toll-like receptor 4

## Abstract

Mollusk hemocyanins have biomedical uses as carriers/adjuvants and nonspecific immunostimulants with beneficial clinical outcomes by triggering the production of proinflammatory cytokines in antigen-presenting cells (APCs) and driving immune responses toward type 1 T helper (Th1) polarization. Significant structural features of hemocyanins as a model antigen are their glycosylation patterns. Indeed, hemocyanins have a multivalent nature as highly mannosylated antigens. We have previously shown that hemocyanins are internalized by APCs through receptor-mediated endocytosis with proteins that contain C-type lectin domains, such as mannose receptor (MR). However, the contribution of other innate immune receptors to the proinflammatory signaling pathway triggered by hemocyanins is unknown. Thus, we studied the roles of Dectin-1, Dectin-2, and Toll-like receptor 4 (TLR4) in the hemocyanin activation of murine APCs, both in dendritic cells (DCs) and macrophages, using hemocyanins from *Megathura crenulata (*KLH), *Concholepas concholepas* (CCH) and *Fissurella latimarginata* (FLH). The results showed that these hemocyanins bound to chimeric Dectin-1 and Dectin-2 receptors *in vitro*; which significantly decreased when the glycoproteins were deglycosylated. However, hemocyanin-induced proinflammatory effects in APCs from Dectin-1 knock-out (KO) and Dectin-2 KO mice were independent of both receptors. Moreover, when wild-type APCs were cultured in the presence of hemocyanins, phosphorylation of Syk kinase was not detected. We further showed that KLH and FLH induced ERK1/2 phosphorylation, a key event involved in the TLR signaling pathway. We confirmed a glycan-dependent binding of hemocyanins to chimeric TLR4 *in vitro*. Moreover, DCs from mice deficient for MyD88-adapter-like (Mal), a downstream adapter molecule of TLR4, were partially activated by FLH, suggesting a role of the TLR pathway in hemocyanin recognition to activate APCs. The participation of TLR4 was confirmed through a decrease in IL-12p40 and IL-6 secretion induced by FLH when a TLR4 blocking antibody was used; a reduction was also observed in DCs from C3H/HeJ mice, a mouse strain with a nonfunctional mutation for this receptor. Moreover, IL-6 secretion induced by FLH was abolished in macrophages deficient for TLR4. Our data showed the involvement of TLR4 in the hemocyanin-mediated proinflammatory response in APCs, which could cooperate with MR in innate immune recognition of these glycoproteins.

## Introduction

Mollusk hemocyanins are extremely large oxygen transport glycoproteins expressed in gastropods (1, 2). Studies have shown that hemocyanins -known as classical T-cell-dependent antigens- inoculated in mammals trigger an immune response that promotes type 1 T helper (Th1) polarization, which generates a beneficial immunological bystander effect (3). Due to these properties, mollusk hemocyanins are used as carriers in antibody development against hapten molecules and peptides, as well as carriers/adjuvants in experimental therapeutic vaccines for cancer (4, 5). By themselves, hemocyanins act as nonspecific immunostimulants in the postsurgical treatment of superficial bladder cancer (6). In addition, hemocyanins elicit complement activation mediated by C1 binding to human natural antibodies (7). The production of these immunomodulators depends on direct protein purification from gastropods, with the most traditionally used hemocyanins for these immunological applications coming from the gastropod *Megathura crenulata*, also known as keyhole limpet hemocyanin [KLH (8)]. Other alternative hemocyanins have emerged, including those obtained from the gastropods *Concholepas concholepas* [CCH (9)] and *Fissurella latimarginata* [FLH (10)] from the Chilean coasts and from *Rapana venosa* from the Black Sea (RvH), previously referred to as *Rapana thomasiana* [RtH (11)], among others (12, 13).

Hemocyanins are extensively used in clinical and biotechnological applications because they do not produce unwanted side effects; however, the mechanisms involved in their immunomodulatory effects are poorly understood (3). Understanding those mechanisms will be crucial to improve the adjuvant and immunostimulant properties of hemocyanins, which would allow the development of better vaccines and immunotherapies (14, 15). Different characteristics have been suggested to explain the immunological properties of hemocyanins, such as their xenogenicity, large size, complex quaternary structure, presence of a quasi-symmetrical arrangement of repetitive epitopes, and glycan moiety content (16–18). All of these glycoproteins fold as large structures, with molecular weights ranging from 4 to 8 MDa. The basic structure comprises ten subunits that self-assemble in pairs to form a hollow cylinder known as decamer, which can interact to form didecamers (17, 19, 20). However, isolated subunits of CCH (21) and RtH (11) also have immunological effects, suggesting that large size and complex quaternary structure are not the only factors involved in the immunogenicity and antitumor effect of these hemocyanins.

In this context, the glycosylation patterns in hemocyanins have an important role in the stability of their quaternary structure, supporting the interaction between the hemocyanin subunits, but these patterns have also been related to their immunomodulatory effects (18, 22, 23). Mollusk hemocyanins are highly glycosylated proteins, reaching up to 9% (w/w) (24). KLH, CCH, and FLH have different N- and O-glycosylation patterns, with mannose-rich and fucose-rich N-glycans being the most abundant types (10, 22). KLH immunization induces cross-reactive antibodies against T antigen due to the presence of oligosaccharides with the terminal moiety Gal(β1-3)-GalNAc in its structure (25). Moreover, sugars on KLH share the structural motif Gal(β1-6)-Man with side chains on liposaccharide (LPS) from pathogens such as *Salmonella typhimurium* and *Klebsiella pneumoniae*, and they also induce cross-reactivity with soluble egg antigens from the parasite *Schistosoma mansoni* based on fucose-shared glycans (26–28). Furthermore, sera from mice immunized with KLH present cross-reactivity with lipoarabinomannan (LAM) from mycobacteria, the polysaccharide capsule of *Cryptococcus neoformans* and LPS from *Escherichia coli* (29). The MALDI-TOF-MS oligosaccharide profile of CCH and FLH revealed abundant diversity in their mannose-rich branched structures (10). Altogether, these data indicate that mollusk hemocyanins possess characteristic and heterogeneous glycosylation patterns and, therefore, could generate different immune responses. Indeed, the effects of mollusk hemocyanins on antigen-presenting cells (APCs) are variable. Hemocyanins stimulate innate immunity by inducing different temporal patterns of proinflammatory cytokine expression in murine peritoneal macrophages, leading to a classically activated macrophage (M1) profile (30). Remarkably, native FLH induces the activation of murine bone marrow-derived dendritic cells (BMDCs) with Th1-inducing cytokine expression and enhances major histocompatibility complex class II (MHC-II) levels (10).

Our current data show that hemocyanins are internalized by APCs through macropinocytosis and receptor-mediated endocytosis in a calcium-dependent manner, which normally leads to the secretion of proinflammatory cytokines. However, this cytokine secretion significantly decreased using deglycosylated FLH, suggesting the participation of specific glycan receptors in this process (10, 30). APCs have been described to recognize pathogen-derived glycans through C-type lectin receptors (CLR) and Toll-like receptors (TLR), among others, which are able to initiate an immune response (31). Mannose receptor (MR), a member of the CLR family, has been identified as a potential receptor for KLH because the maturation of human dendritic cells (DCs) induced by this hemocyanin is partially inhibited by anti-MR blocking antibody (32). In support of this evidence, we performed surface plasmon resonance studies and showed that KLH, CCH and FLH directly interact with human MR with high affinity in a glycan-dependent manner (33). Furthermore, experiments with the human monocytic THP-1 cell reporter line have shown that KLH activates the transcription factor NF-κB and this activity is inhibited by pharmacological inhibitors of Syk or ERK kinases (34). Therefore, we proposed that different innate immune receptors are directly stimulated by the glycan moieties on hemocyanins, giving them a multivalent nature to initiate an immune response. We hypothesized that the CLRs Dectin-1 and Dectin-2 as well TLR4 play a role in the hemocyanin-induced proinflammatory effects. Dectin-1 and Dectin-2 recognize sugars with β-glucan-rich arrangements or mannose-rich branched structures from pathogens such as mycobacteria and *Schistosoma mansoni*, with some of these sugars existing in hemocyanin glycosylations (35–38). TLR4 recognizes mycobacteria and LPS side chains on bacteria such as *Salmonella typhimurium* or *Escherichia coli*; these side chains have also been described to be similar to hemocyanin glycosylations (29, 39–41).

Here we report that hemocyanin-induced proinflammatory effects in murine APCs are independent of Dectin-1 and Dectin-2 despite *in vitro* KLH, CCH, and FLH binding to both chimeric receptors. Additionally, we demonstrate that hemocyanins trigger the TLR signaling pathway in murine APCs and the main receptor involved is TLR4. These findings open the possibility for further studies of a potential cooperation of TLR4 with MR in the hemocyanin-mediated beneficial immune response due to the multivalent nature of these glycoproteins as highly mannosylated antigens.

## Materials and Methods

### Hemocyanins

Hemocyanins from *Concholepas concholepas* (CCH) and *Fissurella latimarginata* (FLH) were provided by Biosonda Corporation (Santiago, Chile). They were isolated and purified as described by De Ioannes et al. (9) and Arancibia et al. (10), respectively, under sterile and endotoxin-free conditions in PBS (0.1 M sodium phosphate, 0.15 M NaCl, pH 7.2). To ensure that the biological effects observed were due to the CCH and FLH component, as indicated Tynan et al. (42), the endotoxin content of hemocyanin preparations was determined using a PyroGene Recombinant Factor C Endotoxin Detection Assay kit (Lonza Group, Walkersville, MD, USA), and was in a range of undetectable to 0.48 EU/mg, respectively. Endotoxin-free hemocyanin (0.5 EU/mg) from *Megathura crenulata* (KLH) in PBS was purchased from EMD Millipore (Billerica, MA, USA). All chemicals were of analytical-grade, and the solutions were prepared using water for human irrigation (Baxter Healthcare, Charlotte, NC, USA or Fresenius Kabi, Bad Homburg, Germany) and filtered through a 0.2 μm membrane filter (Millipore, Billerica, MA, USA). The protein concentration was determined using the Pierce 660 nm Protein Assay Reagent (Thermo Scientific, Waltham, MA, USA) according to manufacturer indications with bovine serum albumin (Thermo Scientific) as the standard.

### Enzymatic and Chemical Hemocyanin Deglycosylation

Enzymatic N-deglycosylation was performed as described by Salazar (43). Briefly, 1 mg of hemocyanin was diluted in 200 μL of glycine-NaOH buffer (130 mM glycine, pH 8.6) and heated at 60°C for 15 min. Solutions were cooled to room temperature, and 2 μL of N-glycosidase F (PNGase F; EC 3.5.1.52) 500 mU (CarboClip, Asparia Glycomics, San Sebastian, Spain) were added. The mixture was incubated for 24 h at 37°C. Chemical deglycosylation was performed as described by Arancibia et al. (44) with modifications. Briefly, hemocyanins (2 mg/mL) were dissolved in PBS (pH 7.2) containing 15 mM sodium periodate (Merck, Darmstadt, Germany) and incubated for 24 h in the dark at room temperature. Next, 25 μL of glycerol were added per 1 mL of solution, and the mixtures were incubated for 24 h in the dark at room temperature. Enzymatically and chemically deglycosylated hemocyanins were dialyzed against PBS (pH 7.2) containing 5 mM MgCl_2_ and 5 mM CaCl_2_, concentrated in Amicon Ultra-15 10K MWCO tubes (Millipore), and filtered through 0.22-μm membrane filters (Whatman, Maidstone, UK).

### Labeling Hemocyanin With Fluorescent Dyes

Hemocyanins (KLH, CCH and FLH) were labeled with DyLight 594 NHS Ester Dye (Thermo Scientific) according to the manufacturer's instructions. Briefly, 10 mg of protein were diluted in 1 mL of 0.2 mM PBS/0.1 M sodium bicarbonate. Then, the dye was added and incubated with the proteins for 1 h at room temperature. The reaction was stopped with 100 μL of hydroxylamine. Finally, the labeled proteins were concentrated and washed at least five times with 5 mL of PBS at 4°C using an Amicon Ultra-4 tube (Millipore). The integrity of the Alexa Fluor 594-labeled hemocyanins was confirmed by SDS-PAGE.

### Mice

C57BL/6 wild-type (WT) mice were obtained from Universidad de Chile (Santiago, Chile) and bred at Biosonda. The study was performed in strict accordance with the Guidelines for the Care and Use of Laboratory Animals of the National Commission for Scientific and Technological Research of Chile (FONDECYT Project 1151337) and the FUCITED Bioethics Committee. C57BL/6 Dectin-1 knock-out (KO) and Dectin-2 KO mice were obtained from Aberdeen University (Aberdeen, UK) and animals were genotypified by PCR (45, 46). Animal experiments were performed using age-matched male mice and conformed to the animal care and welfare protocols approved by UK Home Office (project license 70/8073) in compliance with all relevant local ethical regulations. C57BL/6 Mal KO, C3H/HeN and C3H/HeJ mice were obtained from Trinity College (Dublin, Ireland). Mice were housed in the Bioresources Unit in Trinity College Dublin. Animals were maintained according to the regulations of the European Union and the Irish Department of Health and all procedures performed were conducted under animal license number B100/3321 and were approved by the Trinity College Dublin Animal Research Ethics Committee (Ethical Approval Number 091210).

### Determination of Hemocyanin Binding to Chimeric Receptors by ELISA

Hemocyanin binding to the receptors Dectin-1 and Dectin-2 (Gordon Brown Laboratory, University of Aberdeen), and TLR4 (Novus Biologicals Centennial, CO, USA) was determined by ELISA, as described by Royer et al. with modifications (47). Briefly, polystyrene plates (Thermo Scientific) were treated overnight at 4°C with increasing concentrations of native or deglycosylated hemocyanins (0.5–10 μg/mL in PBS, pH 7.2). For the negative control, the plates were blocked overnight at 4°C with PBS containing 0.2% gelatin (Merck) to establish background binding. Subsequently, the plates were incubated for 2 h at room temperature with chimeric receptors (Dectin-1-Fc 2.5 μg/mL, Dectin-2-Fc 2 μg/mL, and TLR4-Fc 1 μg/ml) diluted in TBS buffer (10 mM Tris-HCl, 10 mM CaCl_2_·2H_2_O, 154 mM NaCl and 0.05% Tween-20; pH 7.5). Then, the plates were incubated for 1 h at room temperature with goat anti-human IgG serum coupled to alkaline phosphatase (ALP; Sigma-Aldrich, St. Louis, MO, USA) diluted 1:1,000 in TBS. TBS washes were performed between each step. Finally, the plates were incubated for 15 min at 37°C with 1 mg/mL p-nitrophenyl phosphate (pNPP; Thermo Scientific) in ALP buffer (100 mM Tris-HCl, 100 mM NaCl, 1 mM MgCl_2_·6H_2_O; pH 9.5), and the optical density was measured at 405 nm in a Synergy HTX ELISA plate reader (Biotek Instruments, Winooski, VT, USA). The positive control ligand zymosan A (Sigma-Aldrich) for Dectin-1, furfurman (InvivoGen, San Diego, CA, USA), a cell wall preparation from *Malassezia furfur* for Dectin-2, and LPS from *Escherichia coli* serotype R515 (Enzo Life Sciences, Farmingdale, NY, USA) for TLR4 were used. Gelatin (Merck) was used as negative control to establish background binding.

### Hemocyanin Uptake by NIH3T3 Cells

NIH3T3 cells overexpressing Dectin-1 or Dectin-2 with FcRγ were used. NIH3T3 cells transfected with empty vector and overexpressing FcRγ alone were used as control cell lines. Briefly, 2 × 10^5^ NIH3T3 cells were incubated with 10 μg/mL Alexa Fluor 594-conjugated hemocyanin for 1 h at 37°C in serum-free media. Cells were then immediately treated with PBS supplemented with 10 mM EDTA and 4 mg/mL lidocaine (Sigma-Aldrich) and washed twice with FACS buffer (PBS with 0.5% BSA, 2% FCS, 2 mM EDTA) and analyzed on a Fortessa flow cytometer (BD Biosciences, San Diego, CA, USA).

### Cell Culture of Bone Marrow-Derived Dendritic Cells (BMDCs)

BMDCs were generated as described by Lutz et al. (48). Briefly, 2 × 10^6^ bone marrow cells from C57BL/6, C3H/HeN, or C3H/HeJ mice were seeded in 10-mm bacteriological culture dishes in RPMI (HyClone, Logan, Utah, USA) medium supplemented with 20 ng/mL mouse recombinant GM-CSF (Miltenyi Biotec, Bergisch Gladbach, Germany) and cultured for 10 days, after which they were collected via gently pipetting in preparation for incubation with hemocyanins. For most experiments, BMDCs were seeded at 1 × 10^6^ cells/mL in 6-well plates and incubated with the hemocyanins (1 mg/mL each) for 24 h. Then, the supernatants were collected to measure the cytokine levels, whereas the cells were treated with PBS supplemented with 10 mM EDTA and 4 mg/mL lidocaine and washed twice with FACS buffer in preparation for the maturation marker analysis by FACS. LPS from *Escherichia coli* Serotype R515 (Enzo Life Sciences) and LPS from *Salmonella enterica* serotype typhimurium (Sigma-Aldrich) and zymosan A (Sigma-Aldrich), were used as positive controls*;* PBS or unstimulated cells were used as a negative controls. The TLR2 control Pam3CysK4 was from Invitrogen Life Technologies (Waltham, MA, USA). The TLR9 control CpG oligonucleotide was from Oligos (Wilsonville, OR, USA).

### Cell Culture of Peritoneal Macrophages and Immortalized Bone Marrow-Derived Macrophages (iBMMs)

Peritoneal macrophages from C57BL/6 mice were prepared according to the method described by Zhang et al. (49), with some modifications. In brief, mice were injected with 1 mL of a sterile solution of thioglycollate-enriched medium. After 4 days, the animals were euthanized and immediately injected with 5 mL sterile ice-cold 5 mM EDTA in PBS into the peritoneal cavity, and the peritoneal fluid was collected. Cells were centrifuged at 1,400 rpm twice for 5 min, and the pellet was resuspended in RPMI medium. For most experiments, peritoneal macrophages were seeded at 1 × 10^6^ cells/mL in 6-well plates and incubated with hemocyanin (1 mg/mL each) for 24 h. Then, the supernatants were collected to determine the cytokine levels. The same controls as listed above were used. iBMMs from C57BL/6 WT and TLR4 KO mice, which were generated using J2 transforming retroviruses (expressing Raf and Myc), as described previously (50), were a gift from Prof. D. Golenbock (University of Massachusetts Medical Center, USA). iBMMs were seeded at 1.5 × 10^5^ cells/mL in 96-well plates and harvested after 24 h of incubation with 1 mg/mL FLH. The supernatants were assessed for cytokine levels. LPS from *Escherichia coli* serotype R515 (Enzo Life Sciences) was used as a positive control, and PBS was used as a negative control.

### TLR4 Inhibition With Blocking Antibody

The procedure of Morhidan et al. (51) with modifications was used. Briefly, BMDCs and peritoneal macrophages grown as described previously, seeded at a density of 5 × 10^4^ cells in a volume of 100 μL in 96-well plates, were preincubated with 10 μg/mL of the blocking rat anti-mouse anti-TLR4/MD-2 monoclonal antibody MTS510 (Thermo Scientific) or 10 μg/mL of rat IgG2a isotype control (Santa Cruz Biotechnology, Dallas, TX, USA) for 1 h; subsequently, they were stimulated with 1 mg/mL of FLH. As TLR4 agonist positive control, 10 ng/mL LPS from *Escherichia coli* serotype R515 (Enzo Life Sciences) was used. As a negative control, unstimulated cells were used. After 24 h of stimulation, supernatants were assessed for IL-12 p40 and IL-6 measurements as described below.

### Cytokine Determination

Supernatants from BMDCs, peritoneal macrophages and iBMMs cultures were stored at −20°C until further analyzed. Levels of the murine cytokines IL-12p40 and IL-6 were measured in supernatants in duplicate using commercial kits according to the manufacturer's instructions (BD OptEIA ELISA Set, BD Biosciences).

### FACS Analysis of Maturation Markers

Antibodies used for flow cytometry were obtained from Biolegend (San Diego, CA, USA) unless otherwise indicated and targeted the following proteins: mouse MHC-I haplotype H-2Kb (clone AF6-88.5), mouse CD40 (clone 3/23), mouse CD80 (clone 16-10A1), mouse CD86 (clone GL-1) and MHC-II haplotype I-A/I-E (clone 2G9, BD Biosciences). BMDCs were treated with PBS supplemented with 10 mM EDTA and 4 mg/mL lidocaine and washed twice with ice-cold FACS buffer (0.5% BSA, 2% FCS, 2 mM EDTA in PBS). Cells were incubated cells for 30 min on ice with a FcR blocking antibody (clone 2.4G2, Gordon Brown Laboratory, University of Aberdeen) using 0.5 μL of antibody in 100 μL per assay. Then, the cells were incubated with labeled antibodies (1:100 dilution) for at least 30 min on ice and washed twice with ice-cold FACS buffer. Finally, cells were fixed with 2% paraformaldehyde in PBS and analyzed on a Fortessa flow cytometer (BD Biosciences).

### Pharmacological Inhibition of Syk, ERK1/2, p38, and JNK

For Syk kinase inhibition, BMDCs from WT mice were collected, and seeded at 1 × 10^6^ cells/mL in 6-well plates and pretreated for 30 min with 20 μM piceatannol (Enzo Life Sciences) or 1 μM BAY 61-3606 (Enzo Life Sciences) (34, 52). For MAP kinases inhibition, BMDCs from WT mice were collected, seeded at 1 × 10^6^ cells/mL in 24-well plates and pretreated for 1 h with the following inhibitors: 100 μM FR180204 (Tocris Bioscience, Bristol, UK) for ERK1/2 (53), 100 μM SB203580 (Tocris Bioscience) for p38 or 20 μM SP600125 (Tocris Bioscience) for JNK (54). Then, BMDCs were incubated with 1 mg/mL of each hemocyanin for 24 h, and the supernatants were collected to determine the cytokine levels. The control treatments were performed with the respective DMSO percentage used as a vehicle for most inhibitors. The Alamar Blue® bioassay (Thermo Scientific), a redox indicator of cell viability, was used according to the manufacturer's instructions to assess cell viability in BMDCs cultures with pharmacological inhibitors (55). Under the conditions described above, the cell viability was >80–90%.

### Western Blotting Analysis of Syk and ERK1/2 Activation

First, extracts of 2 × 10^6^ BMDCs cultured with hemocyanins were prepared. LPS or zymosan A were included as positive controls, and PBS and untreated medium were included as negative controls; all cells were treated for the indicated time period. Cell lysates were prepared with RIPA buffer supplemented with protease and phosphatase inhibitors. For Syk kinase analysis, cell lysates were subjected to SDS-PAGE using a 4–12% gradient gel (NuPAGE Novex Tris-Bis, Thermo Scientific) for 1 h at 200 V (56). Then, proteins were transferred onto a PVDF membrane (Thermo Scientific) at 200 mA for 1.5 h as described by Towin et al. (57). The membranes were blocked for 1 h at room temperature with 5% BSA in TBS containing 0.05% Tween-20 (TBS-T) and then incubated with primary antibodies diluted in TBS-T containing 1% BSA overnight at 4°C as follows: anti-phospho-Syk (Y525/Y526) (1:1,000; Cell Signaling Technology, Danvers, MA, USA; clone C87C1), anti-Syk (1:1,000; Abcam, Cambridge, UK; clone SYK-01) and GAPDH (1:5,000; Cell Signaling Technology, clone 14C10). Then, membranes were washed three times with TBS-T and probed for 1 h at room temperature with an anti-rabbit IgG HRP-conjugated antibody (1:5,000; Cell Signaling Technology) diluted in 1% BSA for phospho-Syk and GAPDH and an anti-mouse IgG HRP-conjugated antibody (1:10,000; Jackson ImmunoResearch, West Grove, PA, USA) diluted in 1% BSA for Syk. Membranes were washed and incubated with Femto ECL Western blotting substrate (Thermo Scientific) for protein band detection. For ERK1/2 kinase analysis, cell lysates were subjected to SDS-PAGE using a prepared 12% polyacrylamide gel for approximately 3 h at 80 V. Proteins were transferred onto a nitrocellulose membrane (Thermo Scientific) at 400 mA for 1.5 h. The membranes were blocked with 5% nonfat dry milk in TBS-T for 1 h at room temperature and then incubated overnight at 4°C with primary antibodies targeting the following proteins: phospho-ERK1/2 (Thr202/Tyr204) (1:1,000; Cell Signaling Technology, clone D13.14.4E) and ERK1/2 (1:1,000; Cell Signaling Technology) diluted in TBS-T and GAPDH (1:1,000; Cell Signaling Technology, clone 14C10) diluted in 5% nonfat dry milk in TBS-T. Membranes were washed three times with TBS-T and separately probed for 2 h at room temperature with anti-rabbit IgG HRP-linked antibody (1:5,000; Merck) diluted in 5% nonfat dry milk in TBS-T. Finally, the membranes were washed and incubated with EZ-ECL Western blotting substrate (Biological Industries, Cromwell, CT, USA) to detect the protein bands. The densitometry analyses on Western blotting were assessed using the Un-Scan-It Gel 6.1 program (Orem, Utah, USA).

### Statistical Analysis

The results of the experiments are expressed as the mean ± SEM. Comparisons between two groups were performed using the paired or unpaired Student's *t*-test. Comparisons between three or more groups were performed using two-way ANOVA with Bonferroni posttest. Statistical significance was defined at *p* ≤0.05. Analyses were performed using GraphPad Prism software (La Jolla, CA, USA).

## Results

### Glycans Participate in Hemocyanin Binding to the CLR Dectin-1 and Dectin-2 *in vitro*

**Figure 1:**
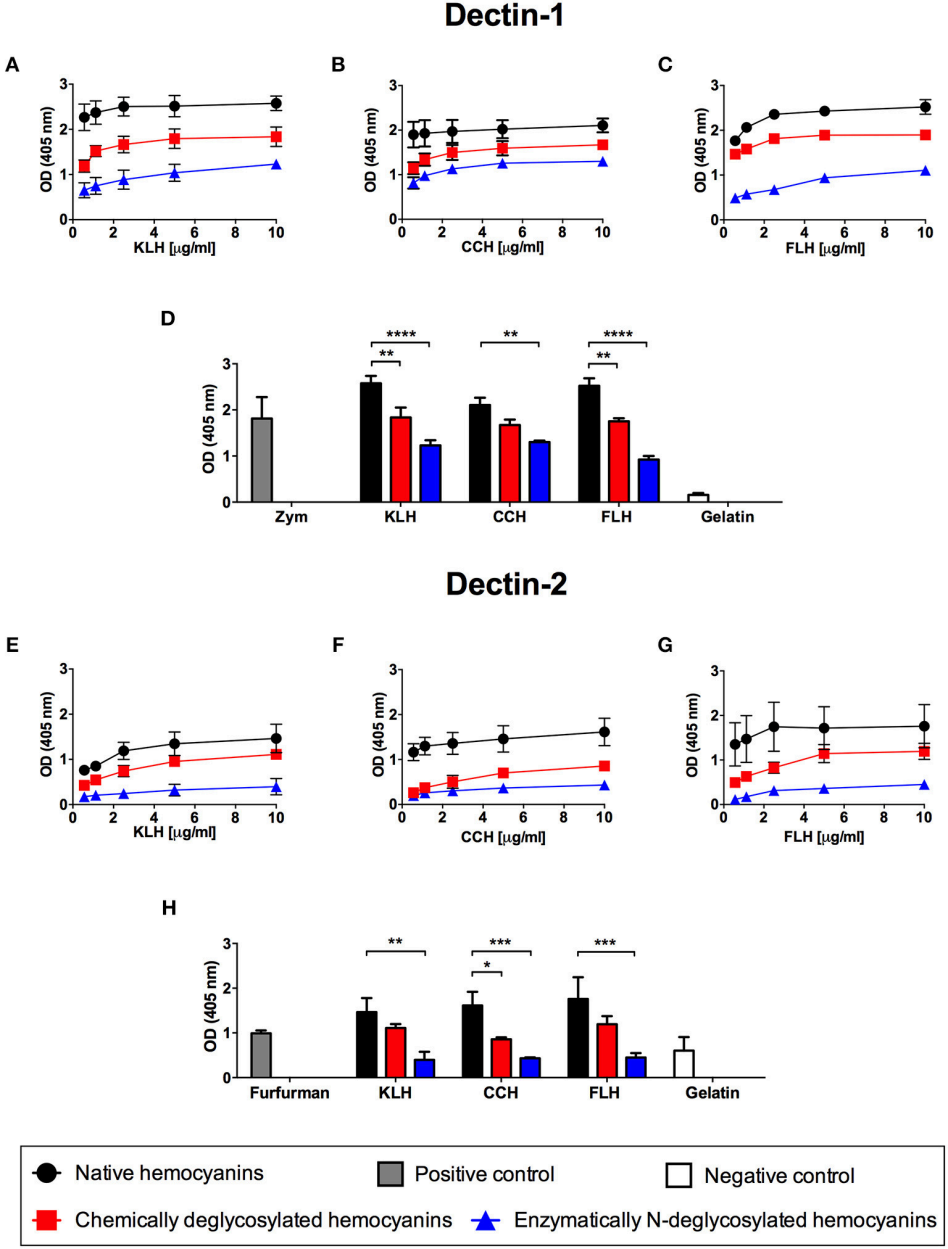
Glycan-dependent binding *in vitro* of hemocyanins to chimeric Dectin-1 and Dectin-2. Indirect ELISA binding curves of KLH **(A)**, CCH **(B)**, and FLH **(C)** to the chimeric Dectin-1-Fc receptor. Polystyrene ELISA plates were incubated with increasing concentrations of native or deglycosylated hemocyanins (0.5–10 μg/mL in PBS, pH 7.2) and then the plates were incubated with chimeric Dectin-1-Fc receptor (2.5 μg/mL) diluted in TBS buffer. Receptor binding was revealed using an anti-human IgG ALP-conjugated antiserum; subsequently, pNPP was added. The plates were read at 405 nm. **(D)** Comparison in the binding of Dectin-1 to native and deglycosylated hemocyanins. The bar graph represents the binding of Dectin-1 to 10 μg/mL of each tested hemocyanin. Zymosan A (Zym) was used as positive binding control and gelatin as negative binding control. Indirect ELISA binding curves of KLH **(E)**, CCH **(F)**, and FLH **(G)** to the chimeric Dectin-2-Fc receptor (2 μg/mL) under the same conditions described above. **(H)** Comparison of the binding of Dectin-2 to native and deglycosylated hemocyanins. The bar graph represents the binding of 10 μg/mL of each tested hemocyanin to Dectin-2. Furfurman was used as a positive binding control and gelatin as negative binding control. For all graphs, black circles represent native hemocyanins, red squares represent chemically deglycosylated hemocyanins, and blue triangles represent enzymatically N-deglycosylated hemocyanins. Gray bars and white bars represent the positive and negative binding control, respectively. Data are presented as the mean ± SEM of two independent experiments. Statistical analyses were performed by two-way ANOVA followed by the Bonferroni posttest. Native hemocyanin vs. chemically or N-deglycosylated hemocyanin: *p < 0.05, **p < 0.01, ***p < 0.001, ****p < 0.0001.

Considering that hemocyanins are highly glycosylated molecules, containing mannose as a more abundant sugar, we propose that the recognition of these glycoproteins by APCs is initially dependent on CLRs, including Dectin-1, the prototypic member of the family, and Dectin-2, which is one of the receptors that can recognize mannose-rich branched structures (35–37). Thus, an indirect ELISA was developed using increasing concentrations of native hemocyanins (0.5–10 μg/mL) to evaluate the binding of Fc chimeric Dectin-1 and Dectin-2 receptors. The results showed that Dectin-1 bound to the three hemocyanins (KLH, CCH and FLH) with similar intensity (Figures 1A–C, respectively; black circles). To demonstrate the dependence of this interaction on glycan, we developed a similar assay using hemocyanins that were chemically deglycosylated by sodium periodate oxidation or enzymatically deglycosylated with PNGase F. Hemocyanins deglycosylated by both methods showed a reduction in binding to Dectin-1, with the lowest binding observed with N-deglycosylated hemocyanins (Figures 1A–C; red squares and blue triangles). The results are summarized in Figure 1D, in which the binding of hemocyanins to Dectin-1 was analyzed at a single representative hemocyanin concentration (10 μg/mL). A significant decrease in the binding of chemically deglycosylated (red bars) and N-deglycosylated (blue bars) with respect to native hemocyanin (black bars) was observed. Similar results were obtained for the binding of Dectin-2 to the three hemocyanins (Figures 1E–G; black circles). Both deglycosylation treatments tended to reduce Dectin-2 binding. However, only N-deglycosylated hemocyanins showed a significant reduction of Dectin-2 binding (Figures 1E–**G**; blue triangles). Similar to the abovementioned experiments, the binding of hemocyanins to Dectin-2 was analyzed at a single representative concentration (10 μg/mL), and a significant effect on N-deglycosylation was observed for all hemocyanins (Figure 1H, blue bars). These results strongly supported the carbohydrate-dependent binding of hemocyanins to Dectin-1 and Dectin-2 *in vitro*, which led us to carry out studies using APCs from Dectin-1 KO and Dectin-2 KO mice.

### The Hemocyanin-Mediated Proinflammatory Response Is Independent of Dectin-1 and Dectin-2

**Figure 2:**
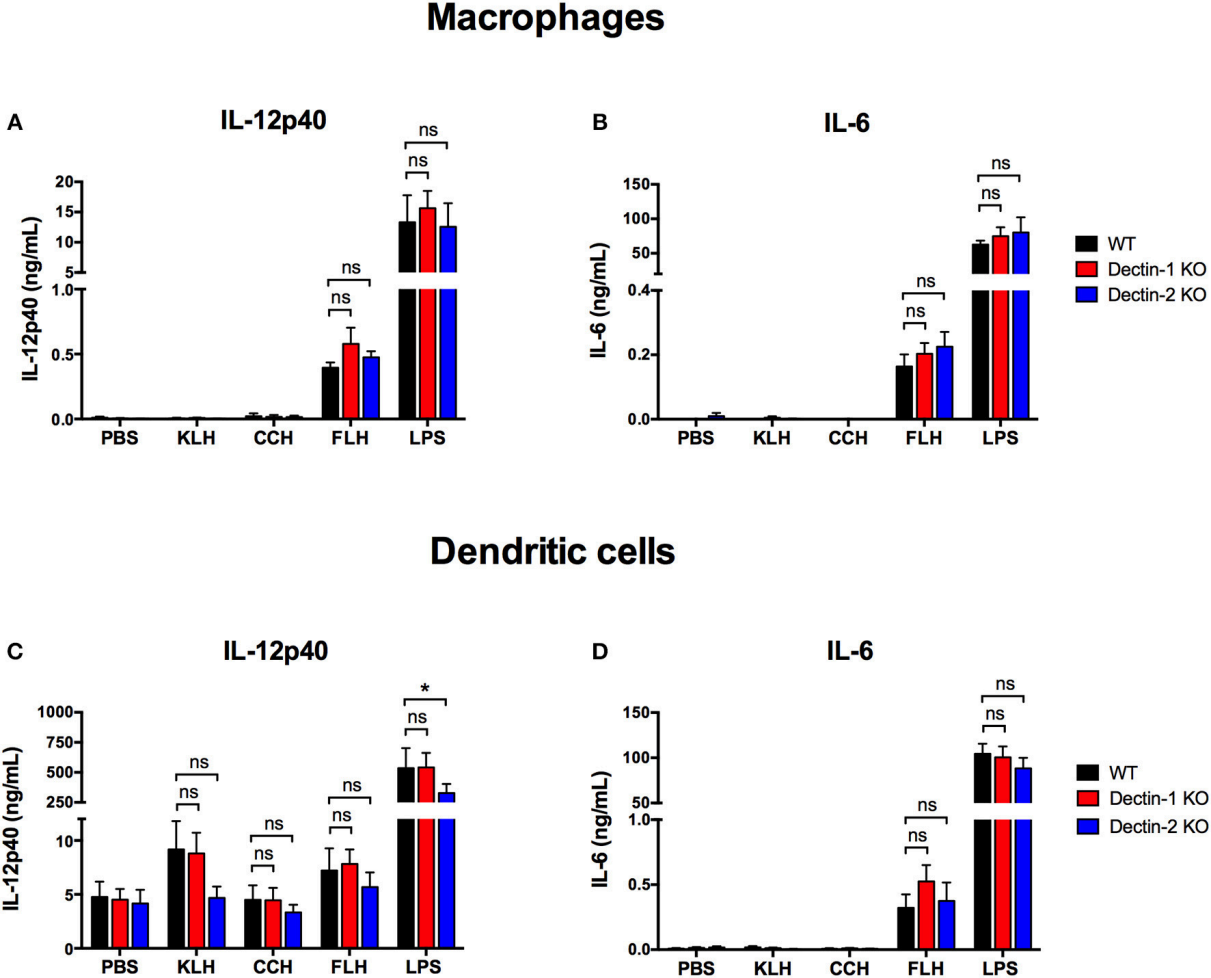
Proinflammatory cytokine secretion induced by hemocyanins is independent of Dectin-1 and Dectin-2. Assessment of IL-12p40 **(A)** and IL-6 **(B)** secretion from peritoneal macrophages from WT (black bars), Dectin-1 KO (red bars) and Dectin-2 KO (blue bars) mice treated with PBS, 1 mg/mL KLH, 1 mg/mL CCH, 1 mg/mL FLH or 1 μg/mL LPS (from *Salmonella enterica*) for 24 h. Supernatants were collected and analyzed by ELISA for cytokine secretion. Assessment of IL-12p40 **(C)** and IL-6 **(D)** secretion from BMDCs from WT (black bars), Dectin-1 KO (red bars) and Dectin-2 KO (blue bars) mice treated for 24 h with the same stimuli described in **(A,B)**. Supernatants were collected and analyzed by ELISA for cytokine secretion. Data are presented as the mean ± SEM of at least three independent experiments. Statistical analyses were performed by two-way ANOVA followed by the Bonferroni posttest. WT vs. Dectin-1 KO or Dectin-2 KO: *p < 0.05; ns = not significant.

Next, we investigated whether the proinflammatory effects of hemocyanins on APCs, such as proinflammatory cytokine secretion and maturation, were associated with Dectin-1 or Dectin-2. Therefore, APCs from Dectin-1 KO and Dectin-2 KO mice that had been previously genotypified were cultured with each hemocyanin for 24 h to analyze IL-12p40 and IL-6 secretion as well as expression of maturation markers (i.e., CD40, CD80 and CD86) and MHC-I and MHC-II upregulation. In peritoneal macrophages from both Dectin-1 KO and Dectin-2 KO mice, IL-12p40 and IL-6 production was comparable to that in cells from WT mice (Figures 2A,B, respectively). No difference was observed for hemocyanins in the case of IL-12p40 secretion from Dectin-1 KO BMDCs (Figure 2C). Likewise, a decreasing trend in IL-12p40 secretion was observed after KLH and FLH treatment in Dectin-2 KO BMDCs compared with the secretion from WT BMDCs. Additionally, LPS treatment induced a significant decrease in IL-12p40 secretion from Dectin-2 KO BMDCs compared with WT BMDCs (Figure 2C). Moreover, IL-6 secretion from Dectin-1 KO and Dectin-2 KO BMDCs was similar to that from WT BMDCs for all hemocyanin treatments (Figure 2D).

**Figure 3:**
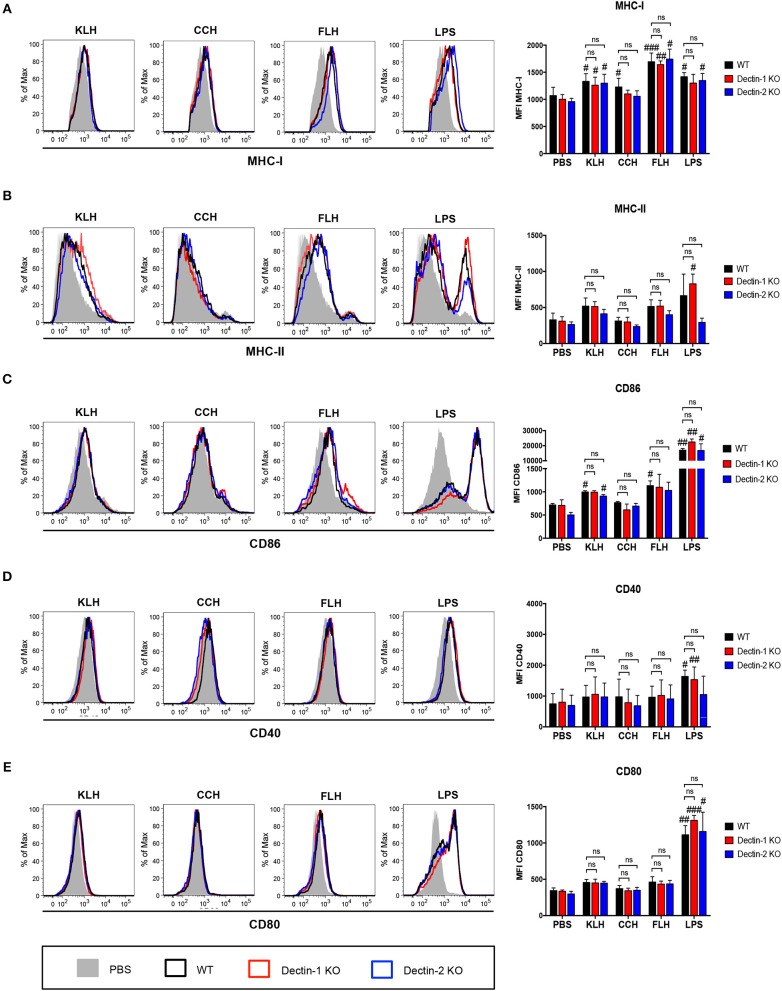
Upregulation of maturation markers induced by hemocyanin treatment is independent of Dectin-1 and Dectin-2. BMDCs from WT (black bars), Dectin-1 KO (red bars) and Dectin-2 KO (blue bars) mice treated with PBS, 1 mg/mL KLH, 1 mg/mL CCH, 1 mg/mL FLH or 1 μg/mL LPS (from *Salmonella enterica*) for 24 h and then analyzed by FACS. **(A)** Representative histograms of MHC-I (H-2Kb) expression after each treatment. The MHC-I mean fluorescence intensity is presented as the mean ± SEM of four independent experiments. **(B)** Representative histograms of MHC-II (I-Ad and I-Ed) expression after each treatment. The MHC-II mean fluorescence intensity is presented as the mean ± SEM of three independent experiments. **(C)** Representative histograms of CD86 expression after each treatment. The CD86 mean fluorescence intensity is presented as the mean ± SEM of two independent experiments. **(D)** Representative histograms of CD40 expression after each treatment. The CD40 mean fluorescence intensity is presented as the mean ± SEM of two independent experiments. **(E)** Representative histograms of CD80 expression after each treatment. The CD80 mean fluorescence intensity is presented as the mean ± SEM of three independent experiments. Statistical analyses were performed using the paired or unpaired Student's *t*-test to compare PBS vs. KLH, CCH, FLH, or LPS: #*p* < 0.05, ##*p* < 0.01, ###*p* < 0.001. To compare WT vs. Dectin-1 KO or Dectin-2 KO, two-way ANOVA followed by the Bonferroni posttest was performed: ns = not significant.

Furthermore, KLH and FLH significantly increased MHC-I expression in WT BMDCs compared with negative control (PBS)-treated BMDCs, but this change was not related to either Dectin-1 or Dectin-2 expression (Figure 3A). Similarly, KLH and FLH tended to enhance MHC-II expression in WT BMDCs; however, this increase was equivalent in dendritic cells from both Dectin-1 KO and Dectin-2 KO mice (Figure 3B). Finally, KLH and FLH slightly activated the expression of the costimulatory molecule CD86 in WT BMDCs, and similar effects were observed in Dectin-1 KO and Dectin-2 KO BMDCs (Figure 3C). However, KLH and FLH did not result in an increase in the costimulatory molecules CD40 and CD80 (Figures 3D,E, respectively). In contrast, CCH induced a limited increase in MHC-I relative to that induced by the negative control (PBS) (Figure 3A), but it did not affect MHC-II or the expression of the maturation markers CD86, CD40, and CD80 (Figures 3B– E, respectively). The positive control (LPS) increased the expression of MHC-I and MHC-II as well as the maturation markers in all assessed conditions (Figure 3). These results were further confirmed using a fibroblast cell line NIH3T3 stably transfected with either Dectin-1 or Dectin-2 and FcRγ (58). After 1 h of incubation with hemocyanins labeled with Alexa Fluor 594, cells overexpressing these receptors did not exhibit increased uptake of labeled hemocyanins compared to the respective control NIH3T3 cell lines (Supplementary Figure 1). These data indicate that Dectin-1 and Dectin-2 are not endocytic receptors for hemocyanins. Taken together, these results, show that Dectin-1 and Dectin-2 are not the key receptors responsible for triggering the hemocyanin-mediated proinflammatory effects in APCs. However, these results do not eliminate a role for other Syk-coupled CLRs, and thus we explored whether those glycoproteins could trigger the Syk kinase pathway.

### Hemocyanins do not Activate the Syk Kinase Pathway but Trigger the ERK1/2 Kinase Pathway

**Figure 4:**
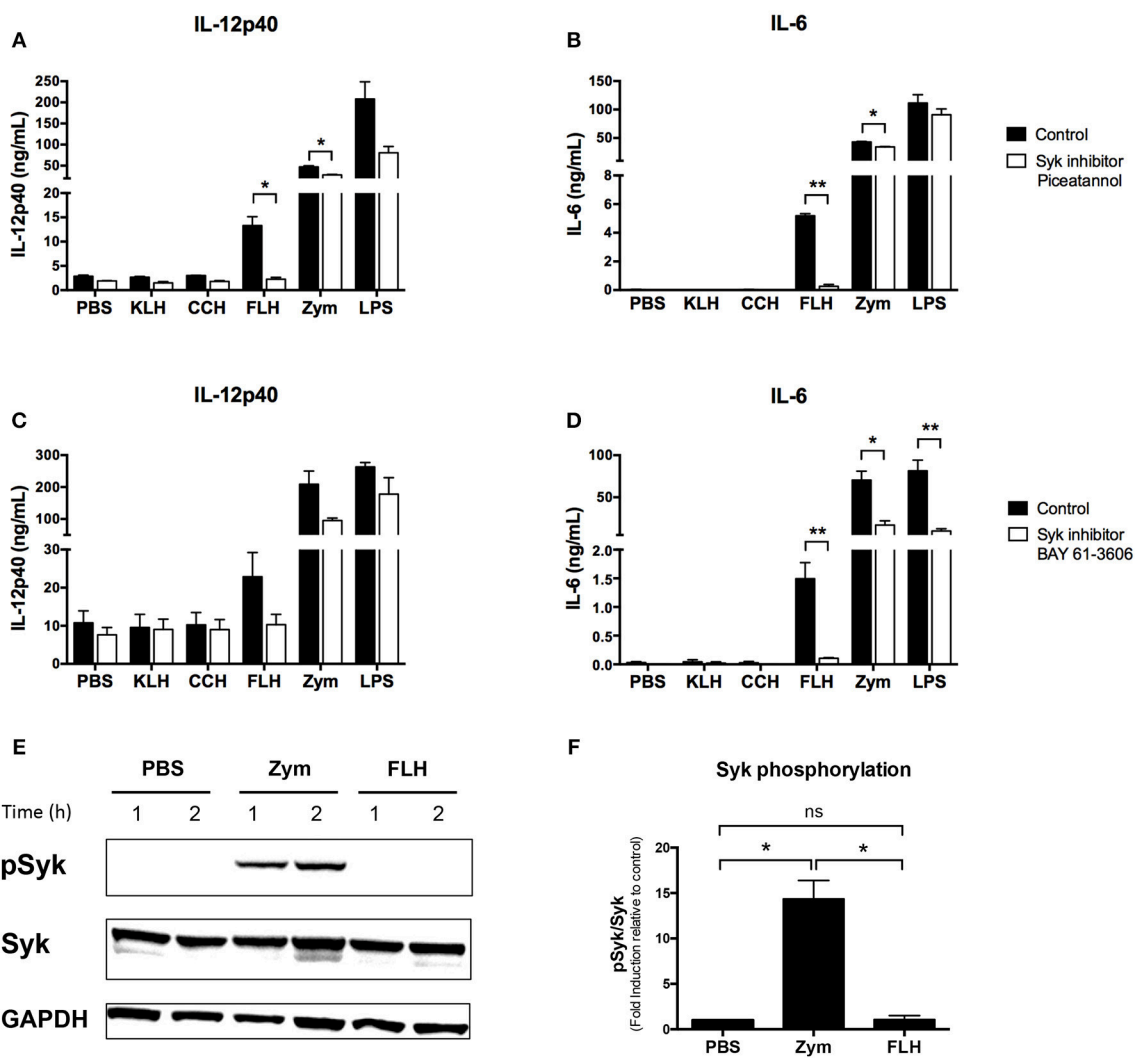
Hemocyanins do not directly activate the Syk kinase pathway in BMDCs. Assessment of IL-12p40 **(A)** and IL-6 **(B)** secretion from BMDCs from WT mice preincubated for 30 min with 20 μM of Syk inhibitor piceatannol or control treatment followed by 24 h of incubation with PBS, 1 mg/mL KLH, 1 mg/mL CCH, 1 mg/mL FLH, 10 μg/mL Zymosan A (Zym) or 1 μg/mL LPS (from *E. coli*). Supernatants were collected, and cytokine secretion was quantified by ELISA. Data are presented as the mean ± SEM of two independent experiments. Assessment of IL-12p40 **(C)** and IL-6 **(D)** secretion from BMDCs from WT mice preincubated for 30 min with 1 μM of the Syk inhibitor BAY 61-3606 or control treatment followed by 24 h of incubation with the same stimuli described above. Supernatants were collected, and cytokine secretion was analyzed by ELISA. Data are presented as the mean ± SEM of three independent experiments. Statistical analyses were performed using the paired Student's *t*-test. Control vs. Syk inhibitors: *p < 0.05, **p < 0.01 **(E)** Western blotting analysis of Syk kinase phosphorylation (Y525/Y526) in BMDCs from WT mice and incubated with PBS (negative control), 50 μg/mL Zymosan A (Zym; positive control) or 1 mg/mL FLH for the indicated time. Data are representative of two independent experiments with similar results. **(F)**. Densitometry analyses of phospho-Syk (pSyk; expressed as fold induction relative to PBS) after 1 h of incubation with each treatment based on two independent experiments shown in **(E)**. Statistical analyses were performed using the unpaired Student's *t*-test to compare each condition: *p < 0.05, ns = not significant.

To complement our previous results, we investigated the role of Syk kinase in hemocyanin-mediated activation of APCs, so IL-12p40 and IL-6 secretion at 24 h was analyzed in WT BMDCs pretreated with the pharmacologic Syk kinase inhibitors piceatannol and BAY 61-3606. IL-12p40 secretion induced by FLH was practically abrogated in cells treated with piceatannol and partially reduced in those treated with BAY 61-3606 (Figures 4A,C, respectively). Similarly, IL-6 secretion induced by FLH was almost abolished after cotreatment with both inhibitors (Figures 4B,D). As expected, zymosan-induced proinflammatory cytokine secretion was diminished by both inhibitors (Figure 4). Surprisingly, LPS treatment showed a trend toward a decreased proinflammatory cytokine secretion with both inhibitors, with a significant decline in IL-6 levels in cells treated with BAY 61-3606 (Figure 4D). Nevertheless, upon assessment of Syk kinase phosphorylation via Western blot, we observed that FLH treatment of WT BMDCs did not induce Syk kinase activation acutely, unlike treatment with the positive control zymosan (Figure 4E). These results were assessed using densitometry image analysis (Figure 4F) and were replicated over shorter stimulation times with FLH as well as with KLH by Western blot and FACS (data not shown). The data showed that Syk kinase inhibition impaired proinflammatory cytokine secretion, but no Syk kinase phosphorylation was detected after hemocyanin treatment of APCs. Taken together, these findings suggest that hemocyanins do not directly activate Syk-coupled CLRs, supporting the stimulation of alternative signaling pathways such as those mediated by TLRs.

**Figure 5:**
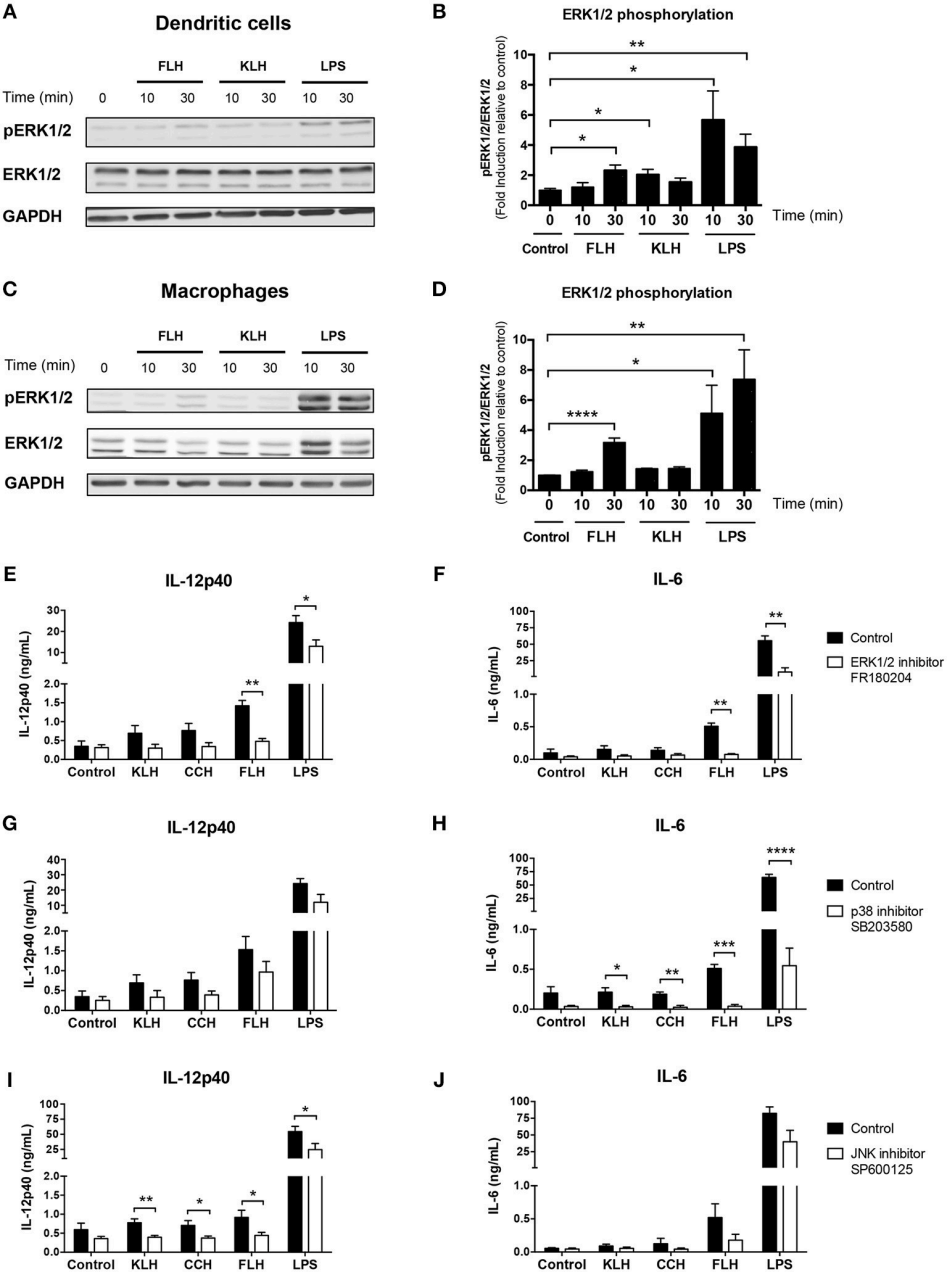
MAP kinase pathways are involved in hemocyanin-induced APC activation. **(A)** Western blotting analysis of ERK1/2 kinase phosphorylation (T202/Y204) in BMDCs from WT mice incubated with 1 mg/mL FLH or 1 mg/mL KLH for the indicated time. Unstimulated cells were used as a negative control and 100 ng/mL LPS (from *E. coli*) as positive control. Data are representative of three independent experiments with similar results. **(B)** Densitometry quantification of phospho-ERK1/2 (pERK1/2; expressed as fold induction relative to negative control) of three independent experiments shown in **(A)**. Statistical analyses were performed using the unpaired Student's *t*-test to compare the negative control vs. treatment: *p < 0.05, **p < 0.01. **(C)** Western blotting analysis of ERK1/2 kinase phosphorylation (T202/Y204) in peritoneal macrophages from WT mice incubated under the same conditions described in **(A)**. Data are representative of three independent experiments with similar results. **(D)** Densitometry quantification of phospho-ERK1/2 (pERK1/2; expressed as fold induction relative to negative control) of three independent experiments shown in **(C)**. Statistical analyses were performed using the unpaired Student's *t*-test to compare the negative control vs. treatment: *p < 0.05, **p < 0.01, ****p < 0.0001. Assessment of IL-12p40 **(E)** and IL-6 **(F)** secretion by BMDCs from WT mice preincubated for 1 h with 100 μM of ERK1/2 inhibitor FR180204 or control treatment, followed by 24 h of incubation with 1 mg/mL KLH, 1 mg/mL CCH, 1 mg/mL FLH or 100 ng/mL LPS (from *E. coli*). Unstimulated cells were used as a negative control. Supernatants were collected, and cytokine secretion was quantified by ELISA. Data are presented as the mean ± SEM of four independent experiments. Assessment of IL-12p40 **(G)** and IL-6 **(H)** secretion by BMDCs from WT mice preincubated for 1 h with 100 μM of p38 inhibitor SB203580 or control treatment, followed by 24 h of incubation under same conditions described in **(E,F)**. Supernatants were collected, and cytokine secretion was quantified by ELISA. Data are presented as the mean ± SEM of four independent experiments. Assessment of IL-12p40 **(I)** and IL-6 **(J)** secretion by BMDCs from WT mice preincubated for 1 h with 20 μM of JNK inhibitor SP600125 or control treatment followed by 24 h of incubation under same conditions described in **(E,F)**. Supernatants were collected, and cytokine secretion was quantified by ELISA. Data are presented as the mean ± SEM of five independent experiments. Statistical analyses were performed using unpaired Student's *t*-test to compare control vs. MAP kinase inhibitors: *p < 0.05, **p < 0.01, ***p < 0.001, ****p < 0.0001.

In contrast, previous data have shown that KLH triggers ERK1/2 phosphorylation in a human monocytic cell line (34). Therefore, we conducted Western blotting analyses as well as those using the selective ERK pharmacologic inhibitor FR180204 to investigate whether hemocyanins could activate the ERK1/2 kinase pathway in BMDCs and peritoneal macrophages from WT mice. We observed a significant induction of ERK1/2 phosphorylation in BMDCs after an acute incubation with FLH at 30 min and KLH at 10 min (Figure 5A). Additionally, significant induction was observed in macrophages incubated with FLH at 30 min (Figure 5C), suggesting the involvement of TLR in the response to hemocyanin-mediated activation of APCs. These results were assessed using a densitometry image analysis (Figures 5B,D, respectively). Moreover, a significant reduction in IL-12p40 and IL-6 secretion induced only by FLH was observed when BMDCs were cultured with the ERK inhibitor FR180204 with respect to the control (Figures 5E,F, respectively). In addition, the effect of pharmacologic inhibitors on other MAP kinases was analyzed, such as p38 mitogen-activated protein kinase (p38) and c-Jun N-terminal kinase (JNK). As shown in Figures 5G,H, the SB203580 inhibitor of p38 decreased the secretion of IL-6 induced by hemocyanins. Likewise, a significant decline in the secretion of IL-12p40 was observed with the JNK inhibitor SP600125 for all hemocyanins (Figures 5I,J, respectively). Collectively, these results suggest that the TLR pathways participate in the hemocyanin-mediated proinflammatory response because ERK1/2, p38 and JNK are directly involved in the signaling pathway of several Toll-like receptors such as TLR4 (59).

### Hemocyanin Binding *in vitro* to TLR4 Is Glycan-Dependent

**Figure 6:**
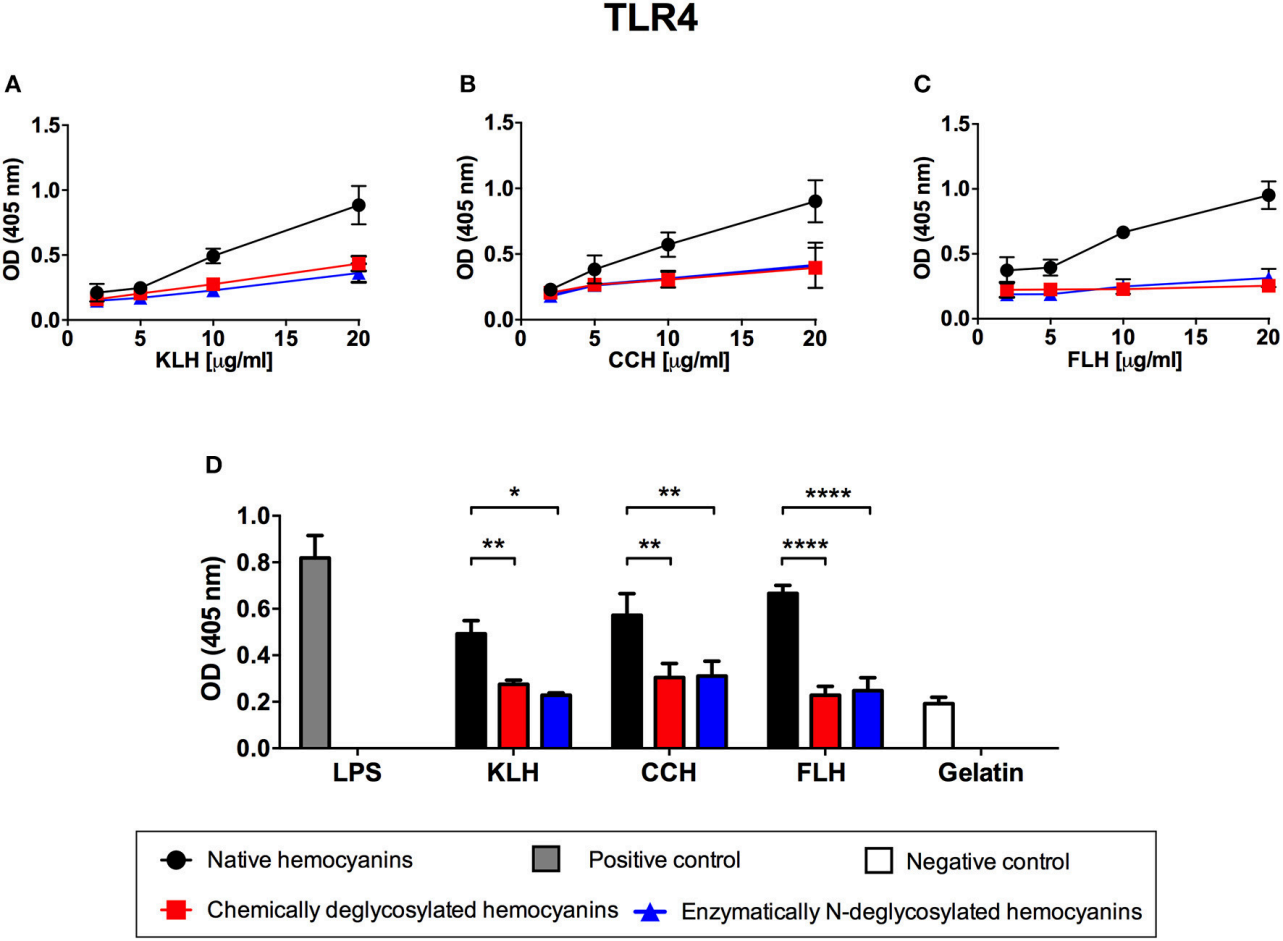
Glycan-dependent binding of hemocyanins to TLR4 *in vitro*. Indirect ELISA binding curves of KLH **(A)**, CCH **(B)**, and FLH **(C)** to chimeric TLR4-Fc receptor. Polystyrene ELISA plates were incubated with increasing concentrations of native or deglycosylated hemocyanins (1–20 μg/mL in PBS, pH 7.2), and then the plates were incubated with chimeric TLR4-Fc receptor (1 μg/mL) diluted in TBS buffer. Binding was revealed using an anti-mouse IgG ALP-conjugated antiserum; subsequently, pNPP was added. The plates were read at 405 nm. **(D)** Comparison of the binding of chimeric TLR4 to native and deglycosylated hemocyanins. The bar graph represents the binding of TLR4-Fc to 10 μg/mL of each tested hemocyanin. LPS (from *E. coli*) was used as a positive binding control, and gelatin served as negative binding control. Black circles represent native hemocyanins, red squares represent chemically deglycosylated hemocyanins, and blue triangles represent enzymatically N-deglycosylated hemocyanins. Gray and white bars represent the positive binding and negative binding control, respectively. Data are presented as the mean ± SEM of three independent experiments. Statistical analyses were performed by two-way ANOVA followed by the Bonferroni posttest. Native hemocyanin vs. chemically or N-deglycosylated hemocyanin: *p < 0.05, **p < 0.01, ****p < 0.0001.

As a first approximation for TLR4 involvement in hemocyanin recognition, an ELISA similar to the one described above for Dectin-1 or Dectin-2 was performed, using increasing concentrations of native hemocyanins (1 to 20 μg/mL) to evaluate the binding of Fc chimeric TLR4 receptor. The results showed that KLH, CCH and FLH bound to TLR4 in a dose-dependent manner (Figures 6A–C, respectively; black circles). This hemocyanin binding was glycan-dependent because it decreased with chemically (red squares) and enzymatically (blue triangles) deglycosylated hemocyanins. The results are summarized in Figure 6D, in which the binding of hemocyanins to TLR4 was analyzed at a single representative hemocyanin concentration (10 μg/mL). A significant decrease in the binding of chemically deglycosylated (red bars) and N-deglycosylated (blue bars) with respect to native hemocyanins (black bars) was observed. LPS, the canonical agonist of TLR4, was used as positive control. The results showed a glycan-dependent binding of hemocyanins to this receptor *in vitro*. Taken together, these data and the previous results for ERK1/2 activation with FLH suggest a probable role for TLR4 in the hemocyanin-induced proinflammatory effect on APCs. Therefore, in the subsequent experiments, we decided to evaluate TLR4 participation, using only FLH because it is the most potent and consistent hemocyanin in our experimental conditions and its proinflammatory effects in all APCs cultures have been demonstrated (5, 10, 30).

### TLR4 Is Required to Induce Hemocyanin-Mediated Proinflammatory Effects in APCs

**Figure 7:**
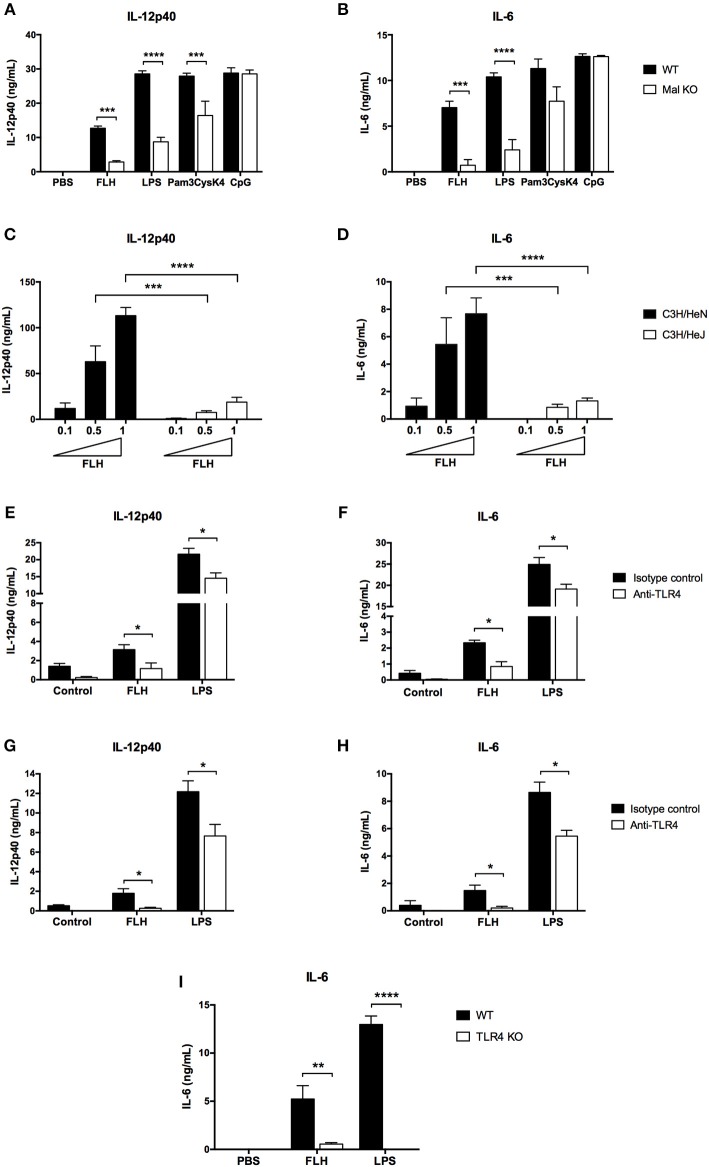
TLR4 participates in hemocyanin-induced proinflammatory cytokine secretion in APCs. Assessment of IL*-*12p40 **(A)** and IL-6 **(B)** secretion by BMDCs from WT and Mal KO mice. BMDCs were incubated for 24 h with PBS, 1 mg/mL FLH, TLR4 control *E. coli* LPS (10 ng/mL), TLR2 control Pam3CysK4 (1 μg/mL) or TLR9 control CpG (4 μg/mL). Cytokine production was analyzed by ELISA. Data are presented as the mean ± SEM of three independent experiments. Statistical analyses were performed by two-way ANOVA followed by the Bonferroni posttest. WT vs. KO: **p < 0.01, ***p < 0.001, ****p < 0.0001. Assessment of IL-12p40 **(C)** and IL-6 **(D)** secretion by BMDCs from C3H/HeN mice (TLR4 WT) and C3H3/HeJ mice (TLR4 mutant). BMDCs were incubated with increasing concentrations of FLH (0.1, 0.5 and 1 mg/mL) for 24 h. Cytokine production was analyzed by ELISA. Data are presented as the mean ± SEM of at least two independent experiments. Statistical analyses were performed by two-way ANOVA followed by the Bonferroni posttest. C3H/HeN vs. C3H/HeJ at the respective FLH concentration: ***p < 0.001, ****p < 0.0001. Blocking anti-TLR4 antibody inhibits IL-12p40 **(E)** and IL-6 **(F)** production in BMDCs. Cells were preincubated for 1 h with 10 μg/mL of anti-TLR4 monoclonal antibody or isotype control, and then 1 mg/mL FLH or 10 ng/mL LPS from *E. coli* was added for 24 h. Cytokine secretion was analyzed by ELISA. Data are presented as the mean ± SEM of three independent experiments. Blocking anti-TLR4 antibody inhibits IL-12p40 **(G)** and IL-6 **(H)** production in peritoneal macrophages. Cells were incubated as described in **(E,F)**. Data are presented as the mean ± SEM of three independent experiments. Statistical analyses were performed using the unpaired Student's *t*-test. Isotype control vs. anti-TLR4 antibody: *p < 0.05. **(I)** IL-6 secretion induced by FLH is impaired in bone marrow-derived macrophages deficient for TLR4. Cells were incubated for 24 h with PBS, 1 mg/mL FLH, and the TLR4 control *E. coli* LPS (10 ng/mL). Then, supernatants were collected and analyzed by ELISA. Data are presented as the mean ± SEM of three independent experiments. Statistical analyses were performed by two-way ANOVA followed by the Bonferroni posttest. WT vs. TLR4 KO: **p < 0.01, ****p < 0.0001.

Since hemocyanins interact with TLR4 *in vitro*, we investigated the involvement of this receptor in the hemocyanin-induced proinflammatory effects in APCs. TLRs signal through several adapter molecules such as MyD88, Mal (MyD88 adapter-like, or also known as TIRAP), TRIF and TRAM to generate a cellular activation response (60). Considering that Mal protein was initially described as a signaling adaptor downstream of TLR4 and TLR2 (61, 62), we assessed whether Mal-deficient BMDCs showed an impaired effect in response to hemocyanins. BMDCs from Mal KO mice were treated with FLH for 24 h, and their cytokine secretion was compared to that of BMDCs from WT mice. We found that IL-12p40 and IL-6 secretion were significantly diminished in Mal KO-derived compared with WT cells (Figures 7A,B, respectively). Moreover, ligands for TLR4 (LPS) and TLR2 (Pam3CysK4) showed an impaired effect in these cells, in contrast to the ligand for TLR9 (CpG), which showed an equivalent effect relative to that of WT cells (Figures 7A,B). These data supported the participation of the TLR pathway in the proinflammatory effects of mollusk hemocyanins in APCs. Since cytokine secretion induced by hemocyanins was impaired in Mal KO BMDCs, we investigated whether TLR4 was required to trigger the hemocyanin-mediated proinflammatory response of APCs. Hence, we established the involvement of TLR4 using BMDCs from C3H/HeJ mice, a strain that carries a specific mutation in the TLR4 gene that impairs its normal function (63). BMDCs derived from C3H/HeJ mice were significantly less stimulated by FLH than those from the control strain C3H/HeN, as IL-12p40 and IL-6 levels were significantly reduced in the BMDCs carrying the mutated TLR4 (Figures 7C,D, respectively). Taken together, these results suggest that TLR4 participates in the hemocyanin-induced proinflammatory effects in antigen-presenting cells.

Thus, to confirm the involvement of TLR4 in the FLH-mediated proinflammatory response, we developed studies with a blocking TLR4 monoclonal antibody on APCs. First, we determined the appropriate concentration of anti-TLR4 antibody to inhibit IL-12p40 and IL-6 secretion induced by FLH in cell culture supernatants (data not shown). Then, APCs were preincubated with anti-TLR4 antibody or an isotype control antibody (10 μg/mL) and subsequently stimulated with FLH; culture medium and LPS were used as negative and positive controls, respectively. The results showed a significant decrease in IL-12p40 and IL-6 secretion in BMDCs (Figures 7E,F) in comparison to the cytokine level of the isotype control antibody. Concomitantly, a significantly reduced level of these cytokines induced by LPS was observed. Similar results were found in peritoneal macrophages (Figures 7G,H). Finally, to confirm a direct role of TLR4 in the FLH-mediated proinflammatory response, we used immortalized bone marrow-derived macrophages from TLR4 KO mice. Accordingly, iBMMs were cultured for 24 h with FLH and then, we analyzed the expression levels of IL-6 in the cell culture supernatants; the same controls described above were used. The results showed that IL-6 secretion was almost abolished compared to that from WT macrophages (Figure 7I). Therefore, these data demonstrated the participation of TLR4 in the proinflammatory response triggered by hemocyanins.

## Discussion

Different studies have proposed that oligosaccharides play a role in the nonspecific immunomodulatory effects of hemocyanins (10, 15, 18, 25). However, to date, there is limited evidence to support this hypothesis. In this respect, we demonstrated that FLH deglycosylation abolished the FLH-induced maturation of murine DCs and attenuated their antitumoral effects in a mouse melanoma model (10). Furthermore, the experiments conducted by Presicce et al. (32) showed that KLH-mediated activation of human DCs was partially inhibited by blocking MR. Moreover, our previous studies showed that KLH, CCH, and FLH bind to human MR *in vitro* with high affinity constants and participate in hemocyanin endocytosis in murine macrophages (30, 33). These data strongly suggest that the innate immune receptors localized on APCs, which bind to sugar moiety-containing proteins such as CLRs and TLRs, can recognize carbohydrate structures on hemocyanins and, in this way, initiate the immune response against them. We hypothesized that APCs recognize hemocyanins as a “highly mannosylated molecular pattern” because of specific glycan moieties that these proteins share with characteristic pathogens (30). Consequently, the aim of this study was to identify new receptors and signaling pathways involved in the beneficial proinflammatory effects induced by KLH, CCH, and FLH in APCs (including dendritic cells and macrophages) from mice. The receptors proposed for this response were the CLRs Dectin-1 and Dectin-2, which are involved in the recognition of cell wall components of fungi (35–38), and TLR4, which is involved in the recognition of bacterial cell wall components (39–41). The main results showed that hemocyanins activated TLR signaling pathways in APCs via TLR4. However, hemocyanins did not activate Syk-coupled CLRs, including Dectin-1 and Dectin-2, in APCs.

The results showed that native hemocyanins bound to chimeric Dectin-1 and Dectin-2 receptors *in vitro* in a glycan-dependent manner. Strikingly, chemical deglycosylation of hemocyanins by periodate oxidation diminished binding to Dectin-1 but not to Dectin-2. We attributed this result to a partial removal of glycans by this method, because other internal sugars could be exposed after treatment, which would explain the residual binding to Dectin-2 (64, 65). Moreover, enzymatic deglycosylation of hemocyanins significantly diminished binding to both receptors. N-deglycosylation with PNGase F achieved almost total abrogation of hemocyanin binding to Dectin-2 but a partial loss binding to Dectin-1, suggesting that O-glycosylations and residual N-glycosylations could be relevant in this recognition. In addition to these results, peritoneal macrophages and BMDCs from mice deficient for Dectin-1 or Dectin-2 showed an equivalent secretion of IL-6 and IL-12p40 and similar upregulation of maturation markers compared with those in WT APCs, confirming that these receptors were not involved in the proinflammatory effects of hemocyanins. Surprisingly, in a cellular context, hemocyanin uptake did not increase in NIH3T3 cell lines overexpressing Dectin-1 or Dectin-2, demonstrating that the binding with chimeric receptors reported above does not trigger hemocyanin endocytosis. A possible explanation for this hemocyanin binding to chimeric Dectin-1 and Dectin-2 is that the ELISA represented the initial exploration to find potential ligands. However, the *in vitro* interaction does not always lead to a response in reporter cell lines or KO cells (66). This inconsistency could be explained by the proper folding of the carbohydrate binding domains in the chimeric protein, as well as the physicochemical conditions of the assay, which could facilitate the intrinsic ligand-receptor interaction in the ELISA. However, this interaction would not occur when the receptor displays its native folding on the plasma membrane, where the binding site could be in a different conformation and even masked by the cellular glycocalyx. In contrast, a significant difference was found only in the secretion of IL-12p40 from Dectin-2 KO BMDCs after LPS treatment (Figure 2C). This result could be explained by the reported cooperation between TLR4 and Dectin-2 (67).

Unexpectedly, we found that KLH and FLH induced an upregulation of MHC-I in BMDCs (Figure 3A), suggesting a role of cross-presentation in the processing of hemocyanins and consequential induction of CD8+ T cells related to the antitumor immune response (68). In this context, it is important to remark that immunohistological studies of biopsies from superficial bladder cancer patients who underwent immunotherapy with KLH showed a massive infiltration of mononuclear cells and CD4+ lymphocytes, as well as, to a lesser extent, CD8+ lymphocytes and granulocytes (69). In addition, during immunotherapy with KLH, a CD4+ lymphocyte response with an increase in CD8+ lymphocytes in the lymphatic nodules has been observed (70). Whether these results can be interpreted in the context of a cross-presentation of KLH has not been addressed. Thus, further studies regarding the role of sugar moieties on hemocyanins in enhanced MHC-I expression as well as the capacity to generate CD8+ T cell-specific responses are necessary to elucidate the antitumor mechanisms of these glycoproteins.

Yasuda et al. (34) suggested that Syk kinase is activated after KLH treatment in the human monocyte cell line THP-1. Nevertheless, these authors did not present data regarding the phosphorylation status of this kinase after KLH treatment, and they only reported an inhibitory effect on NF-κB activation and ERK1/2 phosphorylation after preincubation with the Syk inhibitor BAY 61-3606. Similarly, our data showed that Syk phosphorylation was not detected by Western blot after hemocyanin treatment of APCs (Figure 4E). In contrast, pharmacological inhibition of Syk kinase with piceatannol, a wide spectrum inhibitor, and BAY 61-3606, a more specific inhibitor, diminished the IL-12p40 and IL-6 secretion induced by hemocyanins. An explanation for these apparent contradictory findings is the off-target effects described for both pharmacologic inhibitors used (71–73), implying the limitations of pharmacological tools in the study of signaling pathways. Indeed, we also observed a diminution of LPS-induced proinflammatory cytokine secretion with piceatannol and BAY 61-3606. At this point, it has been previously reported that LPS-mediated activation of APCs is ameliorated by the Syk inhibitor piceatannol, suggesting that the decrease observed in hemocyanin treatments with both inhibitors was a nonspecific inhibition of TLR signaling pathways (74). In addition, we found that pharmacologic inhibition of MAP kinases (ERK1/2, p38, and JNK) impaired hemocyanin-induced proinflammatory cytokine secretion, suggesting a role for TLR pathways. Moreover, we showed that ERK1/2 was phosphorylated upon FLH and KLH stimulation in DCs and macrophages (Figures 5A,C), in agreement with the reported effect of KLH in THP-1 cells (34). Therefore, taken together, these data eliminate Syk-coupled CLRs in the recognition of hemocyanins and suggest the involvement of TLRs. Considering these findings, we evaluated the participation of the TLR signaling pathways in the hemocyanin-induced proinflammatory effect in APCs.

Our interest was focused on TLR4 and the downstream signaling adaptor Mal in APCs because of the interaction of this receptor with glycan patterns expressed by diverse fungi and Gram-negative bacteria, which induces the production of NF-κB-dependent proinflammatory cytokines and strongly drives the immune respond toward Th1 polarization (75). TLR4 recognizes LPS molecules, which exhibit an abundant diversity in their glycan nature (76). LPS contains a highly variable region of O-antigen polysaccharide, similar to the glycans present in hemocyanins (owing to their mannose-rich moieties) (29, 77). TLR4 also participates in the proinflammatory response induced by mannans from *Saccharomyces cerevisiae* and *Candida albicans*, indicating a glycan recognition beyond LPS structure (78). In this regard, we reported a glycan-dependent interaction *in vitro* of hemocyanins with chimeric TLR4 (Figure 6), which suggested as we previously shown, a pivotal role of hemocyanin glycosylations in their beneficial immune responses (10, 43). We also found a Mal-dependent mechanism of APC activation by hemocyanins. Indeed, the functional involvement of TLR4 in the hemocyanin-mediated proinflammatory response in APCs was supported by several lines of evidence, including the use of a specific TLR4 blocking monoclonal antibody, by BMDCs from C3H/HeJ mice, which possess a nonfunctional mutation in this receptor, and by the evaluation of macrophages deficient for TLR4. The results of these experiments showed that APCs display systematically significant decreases in the levels of IL-12p40 and IL-6 induced by FLH with respect to the controls (Figure 7).

However, previous studies in C3H/HeJ mice have shown that both KLH and CCH exhibit antitumor properties against superficial bladder cancer (79), suggesting the involvement of another receptor other than TLR4 in the antitumor effect of hemocyanins. Mal adaptor protein mediates TLR2 and TLR4 signaling (61, 62, 80). Thus, future experiments should focus on the participation of TLR2 in hemocyanin recognition by APCs. Indeed, TLR2 is involved in host defense against *Cryptococcus neoformans* (81), and antibodies against KLH cross-react with this pathogen (29).

**Figure 8:**
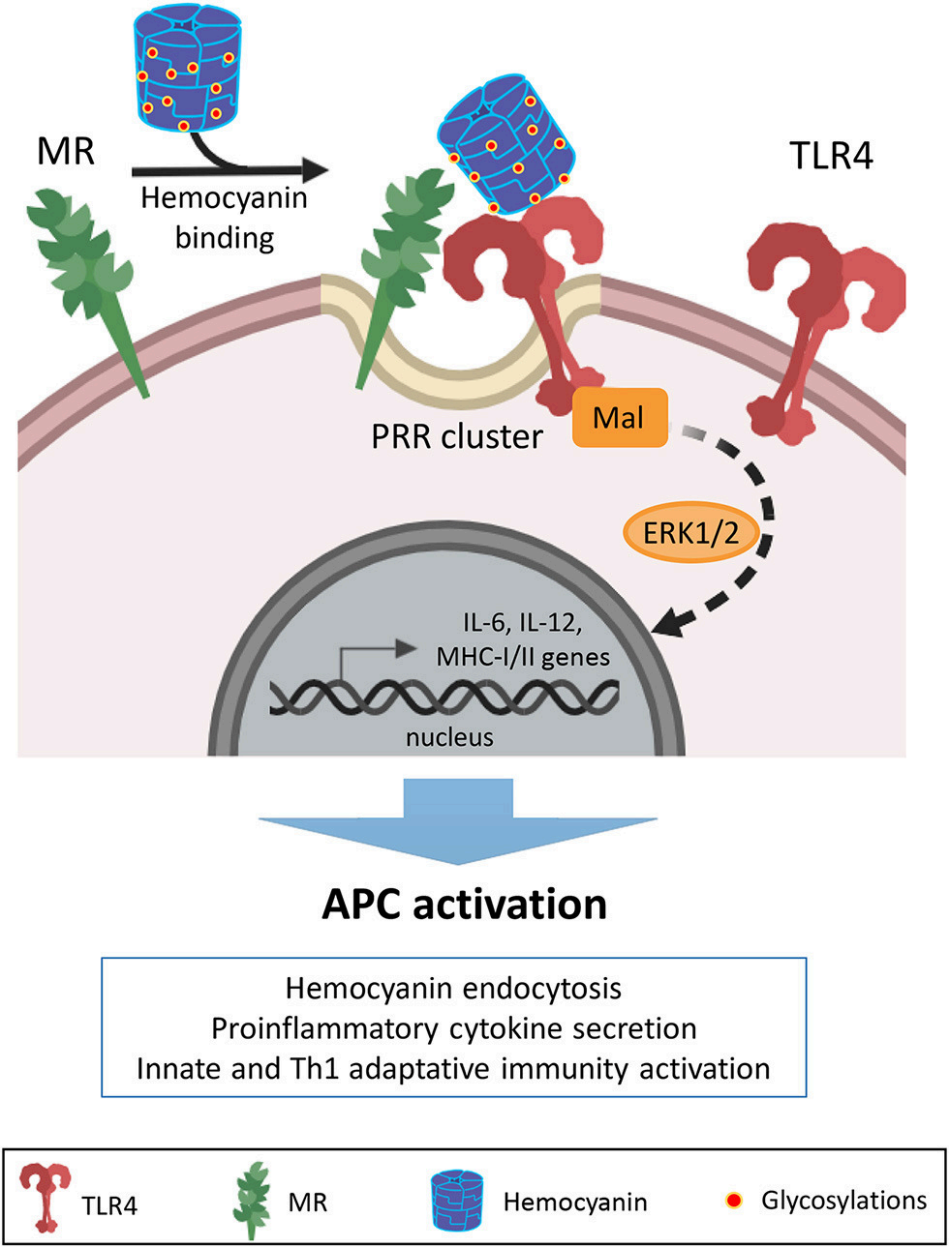
Proposed model of cooperation between MR and TLR4 with regard to the immunostimulatory effects of hemocyanins on APCs. Schematic representation showing a possible mechanism of cooperation between mannose receptor (MR) and TLR4 in the recognition of hemocyanins to initiate a proinflammatory response against these macromolecules. Based on our previous studies, we propose that APCs recognize hemocyanin glycosylations through MR due to their “highly mannosylated molecular patterns”. Then, via clusters of heterologous pattern recognition receptors (PRR) on the APC surface, hemocyanins activate TLR4 to stimulate MAP kinase-dependent downstream signaling pathways. Thus, the cooperation between MR and TLR4 could contribute to the described proinflammatory effects and consequent APC activation induced by hemocyanins.

Remarkably, TLR4 and MR cooperation to trigger intracellular signaling pathways for cytokine secretion in response to different mannosylated antigens, has been previously described (82–84). Mannose receptor plays a role in the APC response to hemocyanins (30, 32, 33). MR lacks a cytoplasmic domain to transduce the external signal to intracellular pathways, which are required to interact with other canonical pattern recognition receptors to mediate intracellular signaling networks for the generation of a proinflammatory response in APCs (85). Therefore, we hypothesize that hemocyanins can be recognized by various host innate immune receptors due to the multivalent nature of their glycosylations, leading to the activation of several signaling molecules of a downstream target that operate in a complementary manner to induce distinct cellular responses, as has been described for microbial ligands (86). Thus, we propose that the hemocyanin-induced proinflammatory response includes an initial binding to MR and consequent activation of the TLR signaling pathway. In the theoretical cooperative mechanism shown in Figure 8, hemocyanins would activate APCs through the clustering of pattern recognition receptors (PRRs), involving the direct interaction with MR and cooperation with TLR4 to induce signaling pathways that generate a beneficial proinflammatory milieu. These hemocyanin-induced proinflammatory effects in APCs induce a beneficial bystander effect, which directs the immune response toward Th1 polarization.

It is important to note that in the comparison of the hemocyanins used herein, FLH showed the greatest immunological effects, as reflected in the quantitative differences in cytokine secretion by APCs, corroborating previous data showing its superior immunomodulatory activity compared with CCH and KLH (10). Indeed, macrophages stimulated with FLH showed an elevated M1 transcript cytokine profile compared with macrophages stimulated with CCH or KLH, fully supporting the notion that the cytokine gene expression profile and cytokine protein secretion induced by CCH, FLH and KLH are significantly different in intensity and temporality for each one (30). Certainly, together these data support potential mechanisms, but they do not provide evidence concerning the cause of these differences, which could be explained, in part, by the hemocyanin binding to MR and TLR4 with different affinities, depending of the oligosaccharides (number, type, and substitution patterns of outer sugar branches) on the protein, leading to differential production of proinflammatory cytokines. Additionally, the different strains used herein showed variations in the levels of cytokines secretion induced by FLH, despite having the same genes, which probably explains the phenotypic differences (87, 88). These genetic background influences have been reported in different inflammatory processes involving TLR4 (89, 90). In this context, we think that host genetic variations and TLR4 signaling could determine the immune response to FLH via modulation of cytokine production. In support of this idea, the early studies of Cerottini and Unanue (91) showed clear differences in the immune responses of different mouse strains to KLH.

Finally, it is relevant to mention that, although a large and compelling body of evidence has shown the valuable clinical outcomes of hemocyanins, remarkably, the molecular and cellular bases of their immunostimulatory activities are scarcely known. In this context, we believe that the present study represents a significant contribution to the knowledge of the immunostimulatory mechanisms of hemocyanins in mammals. From a basic science perspective, a detailed molecular understanding of the receptors and signaling pathways involved in hemocyanin-induced immunostimulatory processes in mammals, will be required to support new strategies to more effectively boost antitumor immunity in adoptive immunotherapy strategies utilizing these glycoproteins. Indeed, hemocyanins are natural, nontoxic, nonpathogenic immunostimulants that exert their effects as immunopotentiators without the unwanted inflammatory side effects of classical adjuvants that drive cell-mediated immune responses, thus making them ideal for long-term on-going treatments (3, 92–94). For instance, KLH has been approved in the superficial bladder cancer treatment, as alternative to BCG (*Bacillus Calmette–Guérin*) arguing these reasons (6, 95). Although there are more potent adjuvants than hemocyanins, most of them are from Gram negative bacteria and emulsions such as Montanide, their toxicity is perhaps the single most important impediment to introduce them to human use (96, 97). Thus, it is necessary to provide effective and safe alternative adjuvants, such as hemocyanins, whose properties could be partially explained as a carbohydrate-based adjuvant (98), beyond to be a protein-based antigen. Considering that innate immune signals modulate not only the magnitude of the adaptive response but also the quality of this response, hemocyanins as carbohydrate-based adjuvants could act giving a signal at level of antigen recognition and APC activation as a TLR4 agonist (signal 0) (99), acting in a synergistic way with the mannose receptor. Indeed, most specific adjuvants, such as TLR agonists act on signal 0, and indirectly by activating APCs and triggering the secretion of proinflammatory cytokines (signal 1), favoring efficient presentation of the co-administered antigen. In addition, hemocyanins do not target costimulatory molecules (signal 2), so over-stimulation would not be a problem. Remarkably, we have also shown that hemocyanins can be used in combination with other adjuvants, lowering their dosage and reducing their cytotoxicity as the case of AddaVax (5) and saponin QS-21 (94), used to enhance the immunogenicity of cancer vaccines. In conclusion, our data show that TLR4 participates in the proinflammatory effects induced by hemocyanins in APCs of mammals. By contrast, the effects in APCs induced by hemocyanins are independent of the CLRs Dectin-1 and Dectin-2, despite the ability of hemocyanins to bind to both receptors *in vitro*. Thus, the data reported in the present study suggest a potential mechanism by which APCs respond to hemocyanins.

## Ethics Statement

The study with C57BL/6 wild-type (WT) mice was performed in strict accordance with the Guidelines for the Care and Use of Laboratory Animals of the National Commission for Scientific and Technological Research of Chile (FONDECYT Project 1151337) and the FUCITED Bioethics Committee. Experiments whith C57BL/6 Dectin-1 knock-out (KO) and Dectin-2 KO mice were performed conformed to the animal care and welfare protocols approved by UK Home Office (project license 70/8073) in compliance with all relevant local ethical regulations. The experiments with C57BL/6 Mal KO, C3H/HeN and C3H/HeJ mice were maintained according to the regulations of the European Union and the Irish Department of Health and all procedures performed were conducted under animal license number B100/3321 and were approved by the Trinity College Dublin Animal Research Ethics Committee (Ethical Approval Number 091210).

## Author Contributions

JJ and MB: conception and design. JJ, MS, SA, FS, and JO-Q: experiment execution and data acquisition. JJ, MS, SA, JV, FS, GB, EL, LM-P, JO-Q, SL, AM, and MB: data analysis and interpretation. JJ and MB: manuscript writing. All the authors read and approved the final version of this manuscript.

### Conflict of Interest Statement

The authors declare that the research was conducted in the absence of any commercial or financial relationships that could be construed as a potential conflict of interest.

## Supplementary Material

Supplementary Figure 1 Dectin-1 and Dectin-2 are not endocytic receptors for hemocyanins. **(A)** Expression of Dectin-1 and Dectin-2 on NIH3T3 cells confirmed by FACS. Anti-HA antibody was used to detect positive expression, and treatment with secondary antibody alone established background staining. Histograms are representative of two independent experiments with similar results. **(B)** NIH3T3-Dectin-1 and parental control NIH3T3 cells were incubated with 10 μg/mL of each labeled hemocyanin (Hcs-Alexa594: KLH-Alexa594, CCH-Alexa594 and FLH-Alexa594) for 1 h at 37°C. Then, the cells were collected and analyzed by FACS. **(C)** NIH3T3-Dectin-2 + FcRγ and parental control NIH3T3-FcRγ cells were incubated with 10 μg/mL of each labeled hemocyanin (Hcs-Alexa594: KLH-Alexa594, CCH-Alexa594 and FLH-Alexa594) for 1 h at 37°C. Then, the cells were collected and analyzed by FACS. **(D)** MFI quantification of NIH3T3-Dectin-1 uptake shown in **(B)**. Data presented as the mean ± SEM of three independent experiments. **(E)** MFI quantification of NIH3T3-Dectin-2 + FcRγ uptake shown in **(C)**. Data are presented as the mean ± SEM of two independent experiments. Two-way ANOVA followed by the Bonferroni posttest was performed to compare control vs. either Dectin-1 or Dectin-2: ns = not significant.
