# Supplementary figures and images for: TLR4, but Neither Dectin-1 nor Dectin-2, Participates in the Mollusk Hemocyanin-Induced Proinflammatory Effects in Antigen-Presenting Cells From Mammals
Source: Front Immunol. 2019 May 31;10:1136. doi: 10.3389/fimmu.2019.01136 (PMC6554540; doi:10.3389/fimmu.2019.01136)

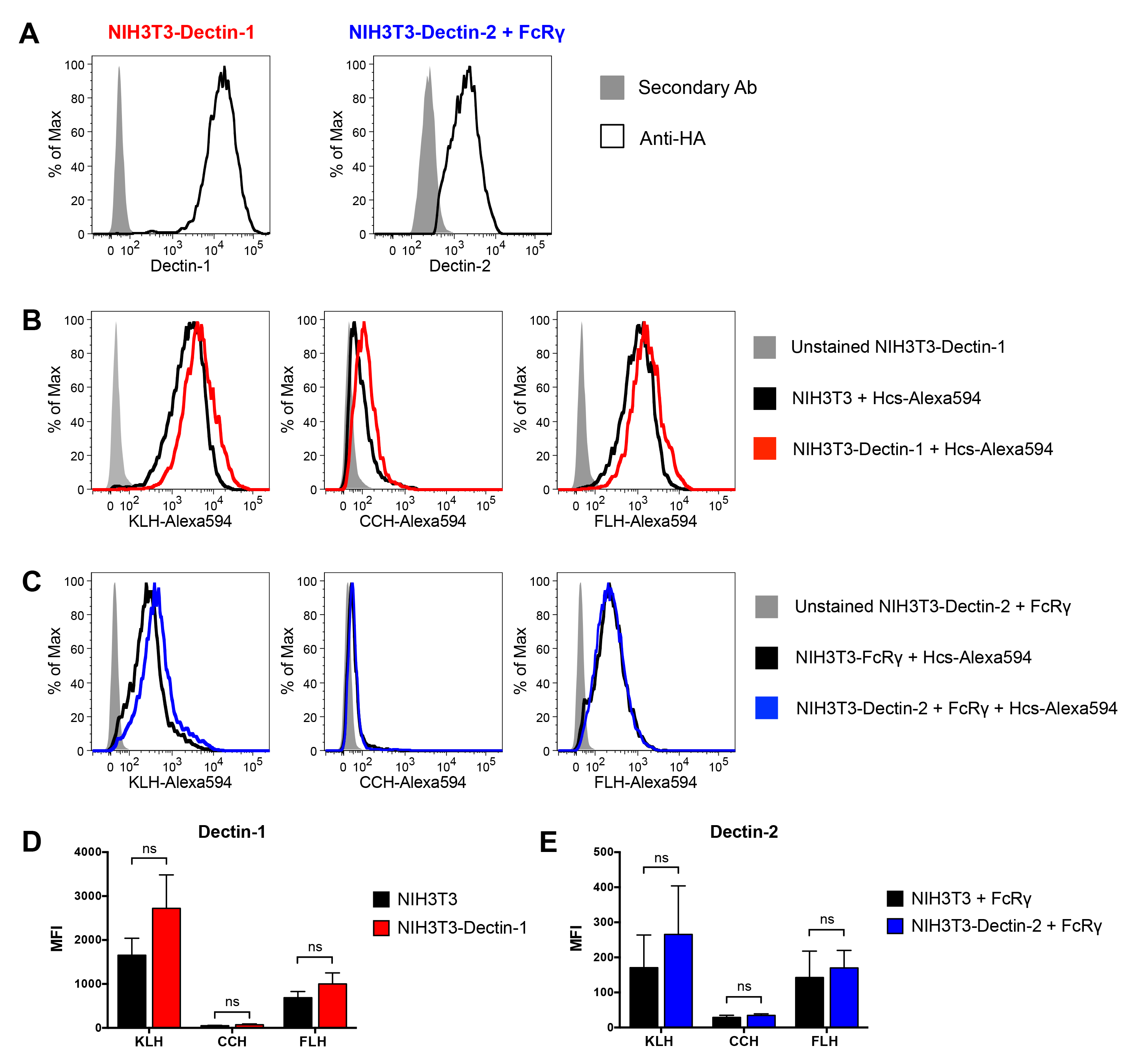

Supplement: Supplementary Figure 1 — Dectin-1 and Dectin-2 are not endocytic receptors for hemocyanins. (A) Expression of Dectin-1 and Dectin-2 on NIH3T3 cells confirmed by FACS. Anti-HA antibody was used to detect positive expression, and treatment with secondary antibody alone established background staining. Histograms are representative of two independent experiments with similar results. (B) NIH3T3-Dectin-1 and parental control NIH3T3 cells were incubated with 10 μg/mL of each labeled hemocyanin (Hcs-Alexa594: KLH-Alexa594, CCH-Alexa594 and FLH-Alexa594) for 1 h at 37°C. Then, the cells were collected and analyzed by FACS. (C) NIH3T3-Dectin-2 + FcRγ and parental control NIH3T3-FcRγ cells were incubated with 10 μg/mL of each labeled hemocyanin (Hcs-Alexa594: KLH-Alexa594, CCH-Alexa594 and FLH-Alexa594) for 1 h at 37°C. Then, the cells were collected and analyzed by FACS. (D) MFI quantification of NIH3T3-Dectin-1 uptake shown in (B). Data presented as the mean ± SEM of three independent experiments. (E) MFI quantification of NIH3T3-Dectin-2 + FcRγ uptake shown in (C). Data are presented as the mean ± SEM of two independent experiments. Two-way ANOVA followed by the Bonferroni posttest was performed to compare control vs. either Dectin-1 or Dectin-2: ns = not significant. [file Image_1.TIFF]
